# Untargeted LC-MS/MS-Based Multi-Informative Molecular Networking for Targeting the Antiproliferative Ingredients in *Tetradium ruticarpum* Fruit

**DOI:** 10.3390/molecules27144462

**Published:** 2022-07-12

**Authors:** Chun-Han Su, Yu-Chieh Cheng, Yu-Chia Chang, Ting-Hsuan Kung, Yu-Li Chen, Kuei-Hung Lai, Hsi-Lung Hsieh, Chun-Yu Chen, Tsong-Long Hwang, Yu-Liang Yang

**Affiliations:** 1 Agricultural Biotechnology Research Center, Academia Sinica, Taipei 115201, Taiwan; 154286@mail.fju.edu.tw (C.-H.S.); j850672002@gmail.com (Y.-C.C.); 2 Research Center for Chinese Herbal Medicine, Graduate Institute of Health Industry Technology, College of Human Ecology, Chang Gung University of Science and Technology, Taoyuan 333324, Taiwan; ycchang03@mail.cgust.edu.tw (Y.-C.C.); oo66931@gmail.com (Y.-L.C.); hlhsieh@mail.cgust.edu.tw (H.-L.H.); 3 Department of Food Science, College of Human Ecology, Fu Jen Catholic University, New Taipei City 242062, Taiwan; 4 Biotechnology Center in Southern Taiwan, Academia Sinica, Tainan 711010, Taiwan; 5 Graduate Institute of Natural Products, College of Medicine, Chang Gung University, Taoyuan 333323, Taiwan; s0911358@gmail.com; 6 Department of Anesthesiology, Chang Gung Memorial Hospital, Taoyuan 333423, Taiwan; 7 PhD Program in Clinical Drug Development of Herbal Medicine, College of Pharmacy, Taipei Medical University, Taipei 110301, Taiwan; kueihunglai@tmu.edu.tw; 8 Graduate Institute of Pharmacognosy, College of Pharmacy, Taipei Medical University, Taipei 110301, Taiwan; 9 Division of Basic Medical Sciences, Department of Nursing, Chang Gung University of Science and Technology, Taoyuan 333324, Taiwan; 10 Department of Neurology, Chang Gung Memorial Hospital, Taoyuan 333423, Taiwan; 11 Graduate Institute of Clinical Medical Sciences, College of Medicine, Chang Gung University, Taoyuan 333323, Taiwan; 12 Department of Chemical Engineering, Ming Chi University of Technology, New Taipei City 243303, Taiwan

**Keywords:** LC-MS/MS, molecular networking, *Tetradium ruticarpum*, Chinese herbal medicine, functional food ingredient, antiproliferative

## Abstract

The fruit of *Tetradium ruticarpum* (TR) is commonly used in Chinese herbal medicine and it has known antiproliferative and antitumor activities, which can serve as a good source of functional ingredients. Although some antiproliferative compounds are reported to be present in TR fruit, most studies only focused on a limited range of metabolites. Therefore, in this study, the antiproliferative activity of different extracts of TR fruit was examined, and the potentially antiproliferative compounds were highlighted by applying an untargeted liquid chromatography–tandem mass spectrometry (LC-MS/MS)-based multi-informative molecular networking strategy. The results showed that among different extracts of TR fruit, the EtOAc fraction F2-3 possessed the most potent antiproliferative activity against HL-60, T24, and LX-2 human cell lines. Through computational tool-aided structure prediction and integrating various data (sample taxonomy, antiproliferative activity, and compound identity) into a molecular network, a total of 11 indole alkaloids and 47 types of quinolone alkaloids were successfully annotated and visualized into three targeted bioactive molecular families. Within these families, up to 25 types of quinolone alkaloids were found that were previously unreported in TR fruit. Four indole alkaloids and five types of quinolone alkaloids were targeted as potentially antiproliferative compounds in the EtOAc fraction F2-3, and three (evodiamine, dehydroevodiamine, and schinifoline) of these targeted alkaloids can serve as marker compounds of F2-3. Evodiamine was verified to be one of the major antiproliferative compounds, and its structural analogues discovered in the molecular network were found to be promising antitumor agents. These results exemplify the application of an LC-MS/MS-based multi-informative molecular networking strategy in the discovery and annotation of bioactive compounds from complex mixtures of potential functional food ingredients.

## 1. Introduction

Functional food ingredients can be obtained from various sources, and Chinese herbal medicine (CHM) serves as one of the important sources of ingredients with therapeutic potential and plays a key role in functional food development and drug discovery. However, CHMs are derived from plants and they are mainly used in the form of crude extracts or raw materials without a well-defined chemical composition, retarding their applications as functional food ingredients [1]. Generally, among various classes of constituents, metabolites are believed to be one of the major ingredients determining the pharmacological properties of CHM [2], and the therapeutic efficacy of preparations may arise from potential synergistic interactions between different metabolites in the CHM materials [3]. Traditionally, the common strategy for CHM metabolite analysis has been bioactivity-guided isolation and identification. However, this approach may lead to re-identification of known compounds without obtaining a comprehensive overview of the metabolite profile. Therefore, a systematic analytical strategy is required to facilitate the process of CHM metabolite mining and analysis for discovering promising functional ingredients.

Untargeted mass spectrometry-based analytical techniques particularly high-resolution liquid chromatography–tandem mass spectrometry (LC-MS/MS) have enabled the large-scale metabolome mining of biological samples including CHMs, and structural information about metabolites can be deduced from the distinct spectral patterns of the mass fragmentation (MS/MS) spectra [4]. However, obtaining metabolite identification and structural elucidation from untargeted MS/MS-based metabolomic profiling still remains challenging. Over the past few years, computational metabolome mining tools and strategies have emerged and started to play important roles in decomposing complex metabolite mixtures [4]. Molecular networking (MN) is a computational strategy for organizing and analyzing MS/MS data; it groups MS/MS spectra based on their spectral similarity, and the molecules with similar fragmentation spectra are connected to form molecular networks [4,5]. Furthermore, the spectral similarities can be extrapolated to the structural similarities between molecules, and therefore the structural relationships among molecules can be visualized [5]. The combination of MN with library matching and other cutting-edge computational tools for network annotation has allowed MN to become a powerful approach in MS/MS-based metabolome mining and metabolite identification [4,6]. In addition, through integrating the sample taxonomy and bioactivity information into the MN, the generated multi-informative molecular networks can further facilitate the elucidation of potentially bioactive compounds in complex mixtures such as CHM [7].

*Tetradium ruticarpum* (A. Juss.) T. G. Hartley is a tree or shrub belonging to the Rutaceae family that is mainly distributed in the temperate and tropical regions of Asia such as China, Japan, and Korea. The fruit of *T. ruticarpum* (TR), also known as Evodiae Fructus or Euodiae Fructus (*wu zhu yu* in Chinese), is a versatile CHM recorded in several ancient and modern Chinese medicine books such as *Shen Nong’s Classic of the Materia Medica* (*shen nong ben cao jing* in Chinese) and the *Taiwan Herbal Pharmacopeia* [8], and it is also used as a medicinal herb in Japan and Korea [9]. According to traditional medical practice, raw TR fruit is usually processed (such as by stir-frying) to improve its function for therapeutic purposes [10]. The fruit of TR is reported to have diverse functions such as “expelling cold” (*san han* in Chinese) and “relieving pain” (*zhi tong* in Chinese), and it can be used alone or in combination with other CHM to treat headache, abdominal pain, vomiting, diarrhea, and menorrhalgia, as well as for modern indications such as irritable bowel disease and dermatitis [9]. TR can serve as a good source of functional ingredients because the crude extracts and bioactive components of the fruit of TR possess various pharmacological properties, such as antiproliferation [11], antitumor [12], anti-inflammation [13], and gastroprotection [14].

To date, more than 160 compounds have been isolated and identified from the fruit of TR, and the types of bioactive components of TR fruit include alkaloids, terpenoids, and phenolics [9]. Among these components, alkaloids are believed to be the main bioactive compounds of TR fruit, and indole alkaloids and quinolone alkaloids are the major types of alkaloids found in TR fruit. Evodiamine and rutaecarpine are two major TR fruit-derived indole alkaloids. Evodiamine has been known to possess therapeutic potential against several cancers such as colon cancer [15], bladder cancer [16], liver cancer [17], and leukemia [18]. Rutaecarpine has been reported to exhibit cardioprotective and vasculoprotective effects such as anti-platelet activation [19] and anti-hyperlipidemia [20]. Furthermore, both evodiamine and rutaecarpine possessed hepatoprotection [21], vasodilatory [22], and anti-inflammatory [23] activities. The quinolone alkaloids of TR fruit such as evocarpine and schinifoline have been reported to exhibit antimicrobial activity [24,25], and schinifoline has also been shown to possess hypoglycemic potential by inhibiting diabetes-related enzymes [26]. Limonin is the major terpenoid of TR fruit with diverse pharmacological activities, such as antibacterial, antivirus, anticancer, and anti-inflammatory properties [27]. Phenolics such as chlorogenic acid and caffeic acid were also found in TR fruit, which mainly possessed antioxidant [28] and anti-inflammatory [29,30] activities.

Previous studies have reported that the antiproliferative activity of TR fruit may be attributed to its alkaloids especially evodiamine [9,11]. Evodiamine can inhibit the proliferation of various human cancer cell lines by inducing apoptosis, cell cycle arrest, or autophagy regulation [12,31]. Although the antiproliferative properties of evodiamine have been widely studied, the antiproliferative activity of other alkaloids and other classes of metabolites in TR fruit remain to be investigated. Therefore, in this study, an untargeted LC-MS/MS-based multi-informative molecular networking strategy was utilized to explore and profile the antiproliferative ingredients in TR fruit.

## 2. Results and Discussion

### 2.1. Antiproliferative Activity of Different Extracts of TR Fruit

The antiproliferative activity of various extracts of TR fruit was determined by measuring their half-maximal inhibitory concentration (IC_50_) for cell growth. Results showed that among 50% EtOH, 50% MeOH, MeOH, EtOAc, and hexane extracts, the EtOAc extract possessed the most potent antiproliferative activity against all tested cell lines, with IC_50_ values of 0.23, 0.32, and 0.47 μg/mL for HL-60, T24, and LX-2 cells, respectively (Table 1). Compared with doxorubicin (a clinical antitumor agent), the EtOAc extract exhibited a similar potency in the growth inhibition of HL-60 cells, while the inhibitory effects of EtOAc extract on T24 and LX-2 cell growth were about 2.5-fold and 4.7-fold weaker than doxorubicin, respectively. In contrast, the 50% MeOH extract exhibited the weakest inhibitory effects on cell growth, with the IC_50_ values close to or greater than 20 μg/mL. These observations indicated that the antiproliferative activity of TR fruit may mainly be attributed to the low-polar components rather than high-polar components. Therefore, to obtain a more specific antiproliferative profile for the low-polar components of TR fruit, the EtOAc extract was selected for further isolation.

Five fractions (F1–F5) of decreasing polarity were collected after the isolation of EtOAc extract by MPLC. The yield of fraction F2 was relatively higher than other fractions, and therefore F2 was subjected to further fractionation to give six subfractions (F2-1–F2-6). The results of cell proliferation assays (Table 1) revealed that F1 had no obvious antiproliferative activity against any of the tested cell lines (IC_50_ > 20 μg/mL), while F2 (IC_50_: 0.80 μg/mL) and F4 (IC_50_: 0.73 μg/mL) showed stronger inhibition of HL-60 cell proliferation than F3 (IC_50_: 2.70 μg/mL) and F5 (IC_50_: 3.25 μg/mL). In the T24 and LX-2 cells, F4 and F5 showed greater inhibition of cell growth than F2 and F3. In addition, the F2-series fractions (F2-1 to F2-6) exhibited diverse antiproliferative effects on different cells, and their IC_50_ values ranged from 0.07 to >20 μg/mL. It was notable that among all EtOAc fractions, F2-3 possessed the most potent antiproliferative activity, with IC_50_ values of 0.07, 0.15, and 0.39 μg/mL for HL-60, T24, and LX-2 cells, respectively, indicating that the major antiproliferative components were enriched in F2-3. F2-3 possessed about 2.4-fold stronger and similar potency as compared with doxorubicin in the inhibition of HL-60 and T24 cell growth, respectively, while the inhibitory effect of F2-3 on LX-2 cell growth was about 3.9-fold weaker than doxorubicin. Furthermore, based on the measured IC_50_ values, all extracts and EtOAc fractions of TR fruit generally exerted stronger antiproliferative activity against HL-60 cells than against T24 and LX-2 cells, implying that the antiproliferative components of TR fruit might be more effective or specific to leukemia cells.

### 2.2. Overview of the Mass Spectral Molecular Network and Targeted Molecular Families of the EtOAc Fractions of TR Fruit

**Figure 1:**
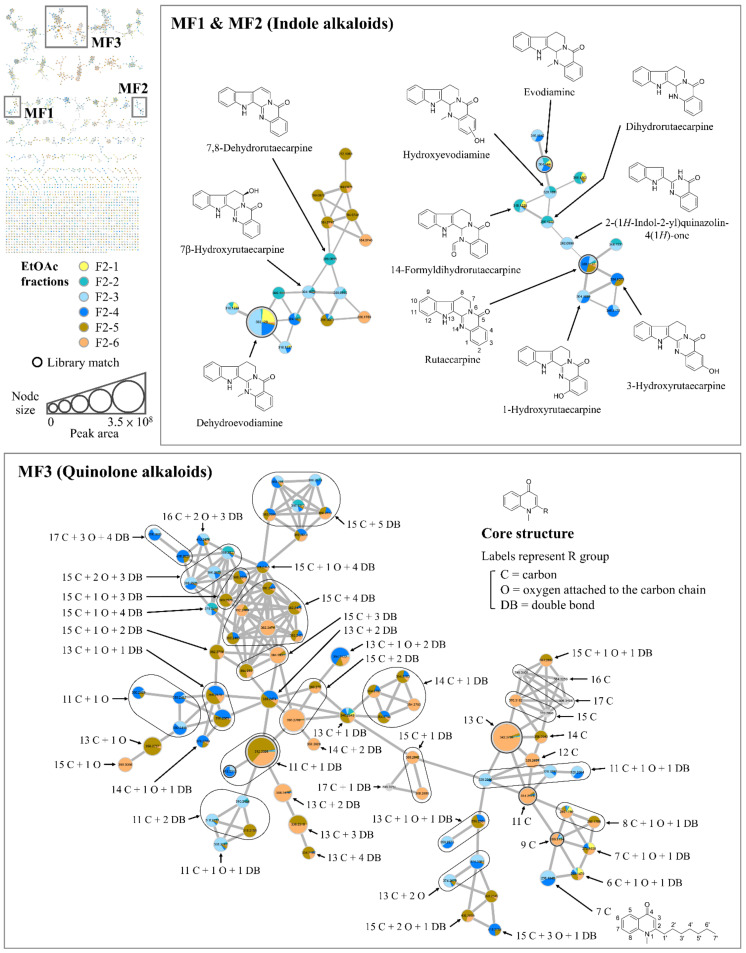
Molecular network of the EtOAc fractions of *T. ruticarpum* fruit and the targeted molecular families (MF) containing indole alkaloids and quinolone alkaloids. Values in the nodes are precursor *m*/*z*. Nodes colored in gray are derived from other EtOAc fractions rather than F2-1–F2-6. The node size is proportional to the sum of the chromatographic peak area. The edge width is proportional to the MS/MS spectral similarity between two connected molecules. The threshold of library match is cosine score ≥0.8.

Because the EtOAc extract of TR fruit possessed stronger antiproliferative activity among different samples, this extract may contain potent antiproliferative compounds, and therefore a mass spectral molecular network was built using the LC-MS/MS data of all fractions of EtOAc extract to facilitate compound analysis, classification, and annotation. Moreover, in order to discover and visualize the characteristic compounds of the most bioactive fraction F2-3, different colors were assigned to the F2-series fractions (F2-1 to F2-6) in the molecular network (Figure 1), and the node size and the pie chart distribution within each node are proportional to the chromatographic peak area.

The results show that the molecular network of the EtOAc fractions of TR fruit contains 2398 nodes (one node represents one molecule) and 191 molecular families (clusters containing ≥2 nodes) (Figure 1, upper left), and the characteristic compounds of F2-3 were mainly distributed into three molecular families (MF) labeled as MF1, MF2, and MF3. Therefore, these molecular families were selected for further investigation. Based on the compound annotation results obtained from spectral library matching and computational tool-assisted structure prediction, numerous indole alkaloids with pentacyclic ring structure were annotated in MF1 and MF2 (Figure 1, upper), while MF3 was composed of quinolone alkaloids possessing a side chain (R group) (Figure 1, bottom). These results are reasonable because indole alkaloids and quinolone alkaloids have been reported to be the main bioactive and characteristic components of TR fruit [9,32,33,34,35].

Although only one indole alkaloid (dehydroevodiamine) was annotated by spectral library matching in MF1, two other known indole alkaloids (7,8-dehydrorutaecarpine and 7β-hydroxyrutaecarpine) of TR fruit were further annotated with the aid of molecular networking (which groups potential structural analogues by comparing the MS/MS spectral similarity), computational tool-based structure prediction, and the information about exact mass and MS/MS fragmentation patterns in the literature [34,36] (Table S1; Supplementary Materials). The structural differences between dehydroevodiamine and the other two indole alkaloids are loss of a methyl group and addition of a hydroxy group or a double bound. On the other hand, the marker compounds of TR fruit, evodiamine, and rutaecarpine, were annotated in MF2 after spectral library matching, and six other indole alkaloids such as dihydrorutaecarpine and 14-formyldihydrorutaecarpine were further annotated similarly based on in silico tool prediction and the literature [9,34,36,37] (Table S1). It was notable that in the bottom of MF2, two connected nodes with similar *m*/*z* values (304.1069 and 304.1075; structural isomers) were both annotated as hydroxyrutaecarpine; although, the exact position of their hydroxy group cannot be determined from MS/MS spectra, the fragment ions at *m*/*z* 136.04 and 169.08 indicated that the hydroxy group is attached to the phenyl moiety of the quinazolinone ring [38]. Along with the information known about hydroxyrutaecarpine isomers (i.e., that a hydroxy group is attached at the *C*1, *C*3, *C*7, or *C*10 position) in TR fruit and the order of their retention time [9,39], the two hydroxyrutaecarpine isomers in MF2 were putatively annotated as 1-hydroxyrutaecarpine and 3-hydroxyrutaecarpine.

MF3 is a relatively large molecular family containing 81 nodes, which are classified as quinolone alkaloids. The basic core structure of the quinolone alkaloids in TR fruit is a 4-quinolone with an *N*-methyl group and an aliphatic side chain (R group) at the α-position of the nitrogen atom (as shown in Figure 1, middle right) [9]. Numerous quinolone alkaloids were clustered together to form MF3 because they possess common characteristic fragment ions with *m*/*z* values of 186.09 and 173.08, resulting from the cleavage of *C*2′–*C*3′ and *C*1′–*C*2′ bonds of the side chain, respectively, and these fragment features also imply the presence of the core structure of quinolone alkaloids [40]. However, due to the potential structural variety of the side chain such as carbon (labeled as C) number as well as oxygen (labeled as O) and/or double bond (labeled as DB) number and position, the exact structure of most quinolone alkaloids in MF3 cannot be determined based on MS/MS spectra, and therefore these compounds were named according to their general structural features of the side chain.

The detailed information such as the precursor *m*/*z* and fragment ions of annotated quinolone alkaloids is summarized in Table S1. Four types of quinolone alkaloids, R = 9 C (1-methyl-2-nonyl-4(1*H*)-quinolone), R = 11 C (1-methyl-2-undecyl-4(1*H*)-quinolone), R = 11 C + 1 DB (1-methyl-2-[(*Z*)-6-undecenyl]-4(1*H*)-quinolone and isomer), and R = 13 C (1-methyl-2-tridecyl-4(1*H*)-quinolone) were first annotated by spectral library matching and served as starting nodes (the nodes with chemical structure information). Next, extended from these starting nodes, various types of quinolone alkaloids were characterized through deducing the numbers of carbon, oxygen, and double bonds in their side chains by comparing the precursor *m*/*z*. For example, the *m*/*z* differences of −2.0157 and +15.9949 indicate the addition of one double bond and one oxygen in the side chain, respectively. Overall, a total of 47 types of quinolone alkaloids were characterized in MF3. Although the exact structures of most quinolone alkaloids remain unclear, their side-chain structural diversity was successfully revealed in MF3, with the numbers of carbon, oxygen, and double bond ranging from 6 to 17, 0 to 3, and 0 to 5, respectively. Notably, 25 types of these characterized quinolone alkaloids possess structural features that have not been found previously in TR fruit. Furthermore, these results also demonstrate the application of the molecular networking strategy in the discovery of novel compounds.

### 2.3. Targeting of the Characteristic Compounds of the Bioactive EtOAc Fraction F2-3 by Multi-Informative Molecular Networking

**Figure 2:**
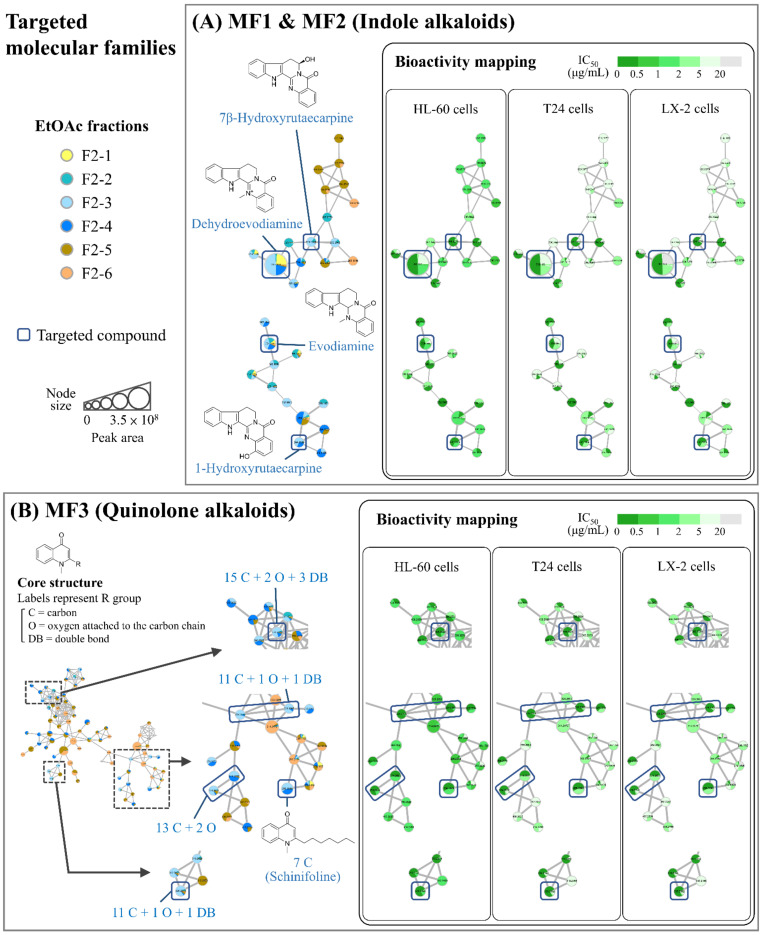
Prioritization of the potentially antiproliferative compounds in *T. ruticarpum* fruit using multi-informative molecular networking: (**A**) Targeted molecular families containing indole alkaloids. (**B**) Targeted molecular family containing quinolone alkaloids. Values in the nodes are precursor *m*/*z*. The node size is proportional to the sum of the chromatographic peak area. The edge width is proportional to the MS/MS spectral similarity between two connected molecules. MF: Molecular family. IC_50_: Half maximal inhibitory concentration.

After visualizing the information about sample taxonomy and compound identity of the F2-series fractions in the targeted molecular families, the antiproliferative activity (level of IC_50_ values) was further integrated to facilitate the prioritization process for exploring potentially antiproliferative compounds in the most bioactive fraction F2-3 (Figure 2). In addition to the multi-informative molecular networking strategy, chromatographic peak area (≥1%) was taken into consideration to aid in targeting the compounds with relatively higher contents in F2-3. Several alkaloids were thereby targeted as the characteristic and potentially antiproliferative compounds in the bioactive fraction F2-3 (Table 2), including four indole alkaloids (evodiamine, dehydroevodiamine, 1-hydroxyrutaecarpine, and 7β-hydroxyrutaecarpine; as shown in Figure 2A) and seven quinolone alkaloids (with four different types of side chain: 7 C (schinifoline), 11 C + 1 O + 1 DB, 13 C + 2 O, and 15 C + 2 O + 3 DB; as shown in Figure 2B). Previous studies have compared the antiproliferative activity of various TR fruit-derived indole and quinolone alkaloids against HL-60 cells, and evodiamine was found to exhibit the strongest activity, while dehydroevodiamine, hydroxyrutaecarpine isomers (1- and 7β-hydroxyrutaecarpine), and most of the quinolone alkaloids possessed moderate inhibitory effects on HL-60 cell growth [32,34]. This preliminarily demonstrates that the multi-informative molecular networking strategy indeed facilitated the prioritization of antiproliferative compounds in the fraction F2-3.

As the targeted compounds belonged to two different classes of alkaloids, two indole alkaloids (evodiamine and dehydroevodiamine) and one quinolone alkaloid (schinifoline), which had higher chromatographic peak areas, were selected as representative compounds for further investigation. The contents of the three selected compounds in the F2-series fractions were measured by LC-triple quadrupole MS. Results showed that among all the F2-series fractions, F2-3 possessed the most abundant evodiamine, dehydroevodiamine, and schinifoline, with contents of 138.7, 92.2, and 16.4 μg/mg, respectively (Table S2; Supplementary Materials). This confirms that these compounds were enriched in the most bioactive fraction F2-3 and may, therefore, serve as the marker compounds of F2-3 and deserve further study to understand their roles in antiproliferative activity.

### 2.4. Individual and Combined Effects of the Targeted Compounds on Cell Viability

The two targeted indole alkaloids (evodiamine and dehydroevodiamine) and one targeted quinolone alkaloid (schinifoline) with higher contents in the bioactive fraction F2-3 were subjected to antiproliferative assays to evaluate the contribution of indole and quinolone alkaloids to the inhibition of cell growth. The IC_50_ values of these alkaloids for cell growth were first determined to compare their antiproliferative activity. The results showed that among these compounds, evodiamine possessed the strongest inhibitory activity, with IC_50_ values of 0.21 and 0.39 μM for HL-60 and LX-2 cells, respectively (Table 3). Compared with doxorubicin, evodiamine exhibited about 1.5-fold stronger and 2.2-fold weaker inhibitory activity in the HL-60 and LX-2 cell growth, respectively. Among the three alkaloid treatments in HL-60 cells, evodiamine exhibited 160-fold and 71-fold stronger antiproliferative activity than dehydroevodiamine (IC_50_: 33.58 μM) and schinifoline (IC_50_: 14.83 μM), respectively. A similar potency was found in the growth inhibition of LX-2 cells, where evodiamine showed 207-fold and 237-fold stronger inhibitory activity than dehydroevodiamine (IC_50_: 80.82 μM) and schinifoline (IC_50_: 92.28 μM), respectively. These observations were consistent with previous studies indicating that evodiamine was a potent antiproliferative compound against various types of cells especially cancer cells [11,41].

Since evodiamine may serve as a structural scaffold with potent antiproliferative activity, it is worthwhile reviewing the neighbor nodes of evodiamine in MF1 (Figure 1, upper right) to find the relevant structural analogues enriched in the bioactive fraction F2-3. Hydroxyevodiamine (the node directly connected to the node of evodiamine in MF1) was thereby first discovered through molecular networking. Among different hydroxyevodiamine isomers, previous studies have revealed that 3-hydroxyevodiamine and 10-hydroxyevodiamine exhibited profound antiproliferative activity against several human cancer cell lines (such as colon cancer HCT116 and breast cancer MDA-MB-435), and several derivatives of 10-hydroxyevodiamine have been synthesized to investigate their structure-activity relationship [42,43]. Some of the derivatives investigated may act as promising antitumor lead compounds. Furthermore, another nearby structural analogue of evodiamine in MF1, dihydrorutaecarpine (the node connected to the node of hydroxyevodiamine), has also been reported to exert strong inhibitory effects on the proliferation of some human cancer cell lines including DU145 (prostate cancer), SKOV3 (ovarian cancer), and U251 (glioma) [44]. These findings demonstrate that the multi-informative molecular networking strategy was useful for exploring the minor compounds with potent bioactivity through targeting the nodes in the potentially bioactive molecular family.

**Figure 3:**
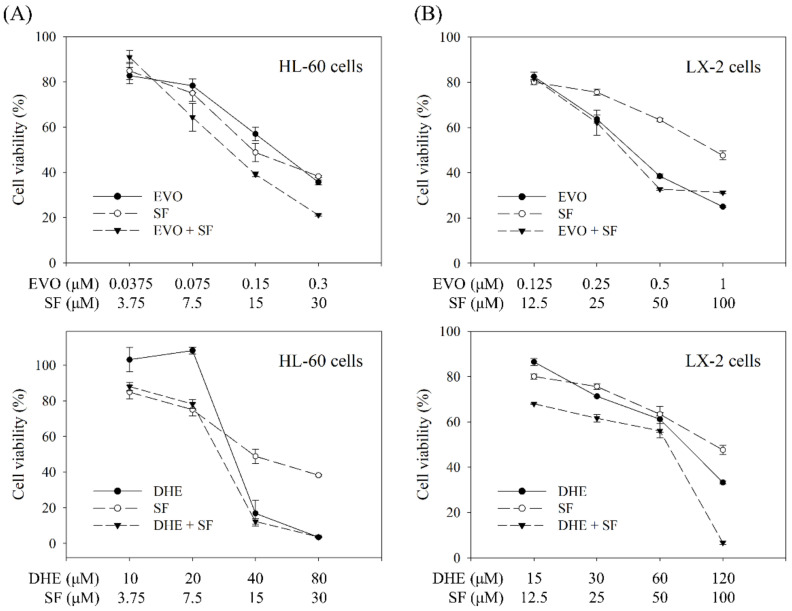
Individual and combined effects of evodiamine (EVO), dehydroevodiamine (DHE), and schinifoline (SF) on the cell viability: (**A**) Inhibitory effects on HL-60 cell growth. (**B**) Inhibitory effects on LX-2 cell growth. Cell viability was measured after 72 h treatment with single compound or the combination of two compounds. Data are presented as mean ± SD (*n* = 3).

With regard to the original targeted compounds, although indole alkaloids and quinolone alkaloids are known as the characteristic compounds of TR fruit [9], the understanding of their potential interactions such as synergistic effects in inhibiting cell growth, remains limited. Therefore, combination treatments using evodiamine (EVO) and schinifoline (SF), as well as dehydroevodiamine (DHE) and SF, were further performed. Figure 3 shows the individual effects of each alkaloid and the combined effects of the two alkaloids used together on the viability of HL-60 and LX-2 cells. In general, all individual treatments led to dose-dependent inhibition of cell growth, except 10 μM and 20 μM of DHE, which had no antiproliferative effect on HL-60 cells. The effects of each combination treatment were quantitatively evaluated by calculating the combination index (CI) using CompuSyn software (version 1.0; ComboSyn, Inc., Paramus, NJ, USA) based on the Chou–Talalay method [45]. CI values < 1, =1, and >1 represent synergistic, additive, and antagonistic effects, respectively. Among the 16 groups of combination treatments, the calculated CI value of the cotreatment of 120 μM DHE and 100 μM SF on LX-2 cells was 0.20, implying that this high-concentration combination inhibited the cell proliferation in a synergistic manner. The LX-2 cell growth was inhibited 66.7% and 52.3% by 120 μM DHE and 100 μM SF, respectively, while up to 93.3% inhibition of cell growth was observed in their cotreatment. However, the calculated CI values of the other 15 cotreatment groups were either close to or greater than one, indicating that no synergistic effect was found in other combination treatments.

Based on the IC_50_ values and the results obtained by individual and combination treatments, evodiamine was verified to be one of the major bioactive compounds contributing to the antiproliferative activity of the bioactive fraction F2-3, and there was no obvious synergistic effect between the tested indole and quinolone alkaloids in the inhibition of cell proliferation.

## 3. Materials and Methods

### 3.1. Materials and Chemicals

The powder of processed fruits of *T. ruticarpum* (TR) was provided by Sheng Chang Pharmaceutical (Taoyuan, Taiwan), and the raw plant materials were authenticated based on the morphological, microscopical, and chemical features described in the *Taiwan Herbal Pharmacopeia* [8]. Evodiamine, dehydroevodiamine, and schinifoline were purchased from ChemFaces (Wuhan, China). Doxorubicin hydrochloride and dimethyl sulfoxide (DMSO; sterile-filtered) were obtained from Sigma-Aldrich (St. Louis, MO, USA). Dulbecco’s modified Eagle medium (DMEM), RPMI-1640 medium, fetal bovine serum (FBS), and antibiotic-antimycotic were purchased from Gibco (Grand Island, NY, USA). Cell Proliferation Reagent (water-soluble tetrazolium salt; WST-1) was obtained from Roche Applied Sciences (Penzberg, Upper Bavaria, Germany). Acetonitrile (LC-MS grade) was purchased from J.T.Baker-Avantor (Radnor, PA, USA). All other chemicals used were of analytical grade.

### 3.2. Extraction and Isolation

The sample was extracted by using an Accelerated Solvent Extractor (ASE 350; Thermo Scientific, Waltham, MA, USA). High pressure was applied by ASE during the extraction process to accelerate and enhance the extraction of organic compounds. Sequential extraction was carried out to extract the compounds with different polarities from the sample, and the extraction solvents and extraction sequence were (1) *n*-hexane, (2) EtOAc, (3) MeOH, and (4) 50% MeOH. In addition, single extraction using 50% EtOH as extraction solvent was also performed. In brief, sample powder (5 g) was packed in a 66 mL stainless steel extraction cell equipped with a cellulose fiber filter at the bottom. After automatic filling of extraction solvent into the cell, the extraction was performed using the following parameters: pressure, 1500 psi; temperature, 50 °C; static extraction time, 15 min; solvent rinse volume, 100%; and nitrogen purge time, 60 s. The products obtained after the extraction process were concentrated and lyophilized to yield hexane, EtOAc, MeOH, 50% MeOH, and 50% EtOH extracts.

The EtOAc extract (177.3 mg) was subjected to a medium pressure liquid chromatography (MPLC) system (Pure C-850 FlashPrep; BUCHI, Flawil, Switzerland) equipped with a C18 cartridge (Acchrom, Beijing, China) to afford five fractions by stepwise water–MeOH–acetonitrile elution: F1 (29.0 mg, elution by 0–70% MeOH for 40 min), F2 (77.4 mg, elution by 80–90% MeOH for 10 min), F3 (5.1 mg, elution by 100% MeOH for 5 min), F4 (28.2 mg, elution by 100% acetonitrile for 5 min), and F5 (6.0 mg, elution by 100% acetonitrile for another 10 min). Fifty milligrams of F2 was further isolated by the same system using gradient elution (0–5 min: 30% MeOH; 5–10 min: 30–70% MeOH; 10–30 min: 70% MeOH; 30–50 min: 70–100% MeOH; 50–55 min: 100% MeOH–100% acetonitrile; and 55–70 min: 100% acetonitrile) to yield six subfractions: F2-1 (8.9 mg, collected from 15 to 20 min), F2-2 (14.8 mg, collected from 20 to 25 min), F2-3 (4.2 mg, collected from 25 to 30 min), F2-4 (3.8 mg, collected from 30 to 40 min), F2-5 (4.5 mg, collected from 40 to 50 min), and F2-6 (2.9 mg, collected from 50 to 60 min).

The greenness assessment of this extraction method was performed by using Analytical GREEnness (AGREE) software [46], and the assessment results are shown in Figure S1 (Supplementary Materials).

### 3.3. LC-MS/MS Analysis

An ultra-high-performance liquid chromatography (UHPLC) system (1290 Infinity II LC system; Agilent Technologies, Santa Clara, CA, USA) coupled to a quadrupole time-of-flight (Q-TOF) mass spectrometer (6545XT AdvanceBio LC/Q-TOF; Agilent Technologies, Santa Clara, CA, USA) was used for LC-MS/MS analysis. Chromatographic separation was performed on an ACQUITY UPLC BEH C18 column (2.1 × 100 mm, 1.7 μm; Waters, Milford, MA, USA) at a flow rate of 0.4 mL/min, and the column oven temperature was set at 40 °C. The mobile phase consisted of water (A) and acetonitrile (B) (both contained 0.1% formic acid). The gradient started from a 5% B isocratic elution for 1 min, followed by a linear gradient of 5% to 99.5% B (1–16 min), and then maintained at 99.5% B for 3 min. The column was re-equilibrated at 5% B for 3 min before the next analysis. The samples were centrifuged at 13,000× *g* for 10 min at 4 °C before subjecting to LC-MS/MS analysis, and the sample injection volume was 10 μL with a concentration of 500 μg/mL.

An electrospray ionization (ESI) source was used and operated in positive ion mode. The source parameters were as follows: capillary voltage, 3000 V; sheath gas flow rate and temperature, 12 L/min and 350 °C; drying gas flow rate and temperature, 9 L/min and 250 °C; nebulizer pressure, 35 psi; fragmentor voltage, 130 V; skimmer voltage, 65 V; and nozzle voltage, 250 V. LC-MS/MS data acquisition was performed using Agilent MassHunter Workstation software (version B.09.00; Agilent Technologies, Santa Clara, CA, USA). Mass spectra were acquired in centroid mode with the *m*/*z* range of 100–1500 in MS1 scans and 50–1500 in MS/MS scans. The scan rate was 0.2 s/spectrum. Automated data-dependent acquisition (DDA) in iterative mode (three consecutive injections of each sample) with active exclusion was performed in MS/MS scans, and the top five most intense precursors were sequentially selected and subjected to collision-induced dissociation with an isolation window of 1.3 *m*/*z* and a collision energy of 40 V.

The intermediate precision and the greenness assessment of this LC-MS/MS method are shown in Table S3 and Figure S1, respectively. The representative LC chromatograms of the EtOAc extract of the TR fruit are provided in the Supplementary Materials (Figures S2 and S3). The LC-MS/MS data are publicly available on the MassIVE website (https://massive.ucsd.edu; accessed on 16 June 2022), and the dataset accession numbers are summarized in Table S4 (Supplementary Materials).

### 3.4. Compound Annotation

The acquired mass spectra were compared against the Traditional Chinese Medicine Personal Compound Database and Library (TCM PCDL; Agilent-NatureStandard) for compound annotation. Further annotation was made by comparing the mass spectra against public mass spectral libraries on the GNPS website (https://gnps.ucsd.edu; accessed on 8 June 2021) [47] or the mass spectra from literature. In silico tools including SIRIUS 4 [48] and MS2LDA [49] were used to predict the compound structure based on mass spectral data. ClassyFire [50] and MolNetEnhancer [51] were used for the structural classification of annotated compounds.

### 3.5. Molecular Networking

#### 3.5.1. LC-MS/MS Data Preprocessing

Raw LC-MS/MS data were converted to .mzML format with MSConvert software (part of the ProteoWizard package; ProteoWizard Software Foundation, Palo Alto, CA, USA) [52] and then preprocessed by using MZmine software (version 2.53; MZmine Development Team) [53]. The mass detection was performed in centroid mode and the noise level was set at 1000 for MS scans and 100 for MS/MS scans. MS chromatograms were built by using the ADAP chromatogram builder with the following settings: minimum group size of scans = 5, group intensity threshold = 1000, minimum highest intensity = 5000, and *m*/*z* tolerance = 0.006 *m*/*z* (or 30 ppm). Chromatographic deconvolution was performed by using the ADAP wavelets algorithm with an S/N threshold of 5, a minimum feature height of 10000, a coefficient/area threshold of 25, a peak duration range of 0.05–2.5 min, and a retention time (RT) wavelet range of 0.03–0.8 min. The *m*/*z* range and RT range for MS/MS scan pairing were set at 0.02 Da and 0.1 min, respectively. The isotopologues were grouped by using the isotopic peaks grouper with an *m*/*z* tolerance set at 0.006 *m*/*z* (or 30 ppm) and an RT tolerance set at 0.2 min. Peak alignment was achieved by using the join aligner, and the parameters were set as follows: *m*/*z* tolerance = 0.006 *m*/*z* (or 30 ppm), RT tolerance = 0.2 min, weight for *m*/*z* = 2, and weight for RT = 1. The missing peaks in the aligned peak list were filled by using the peak finder (multithreaded) with the following parameters: intensity tolerance = 30.0%, *m*/*z* tolerance = 0.005 *m*/*z* (or 20 ppm), and RT tolerance = 0.1 min. After data processing, the obtained results were exported as an .mgf file (containing MS/MS spectral information) and a .csv file (containing a feature list with MS1 *m*/*z* value, peak retention time, and peak area information) by using the “Export/Submit to GNPS-FBMN” built-in function.

#### 3.5.2. Molecular Network Generation Using GNPS

The molecular network was generated based on the preprocessed LC-MS/MS data by using the Global Natural Products Social Molecular Networking (GNPS; https://gnps.ucsd.edu; accessed on 8 June 2021) web platform with the feature-based molecular networking workflow (version 28.2) [47,54]. The parameter settings on the GNPS website were as follows: precursor ion mass tolerance, 0.02 Da; fragment ion mass tolerance, 0.02 Da; minimum pair cosine score, 0.6; and minimum matched fragment ions, 6. The generated molecular network was downloaded as Cytoscape data and then visualized and edited using Cytoscape software (version 3.8.2; Cytoscape Consortium, San Diego, CA, USA) [55].

### 3.6. Quantification of Targeted Compounds

The compounds targeted by the molecular networking strategy were quantified by using a UHPLC (Nexera X2; Shimadzu, Kyoto, Japan) coupled to a triple quadrupole mass spectrometer (LCMS-8045; Shimadzu, Kyoto, Japan) with an ESI source operated in positive ion mode. The conditions of chromatographic separation were the same as described in Section 3.3. The identity of the targeted compounds (evodiamine, dehydroevodiamine, and schinifoline) was confirmed by comparing the retention time and mass spectrum to their respective authentic standards. The contents of the three targeted compounds in the sample were calculated based on the calibration curves of external standards detected in multiple reaction monitoring (MRM) mode, and the MRM transitions were set as follows: evodiamine, *m*/*z* 304.1 → 134.1; dehydroevodiamine, *m*/*z* 302.1 → 286.1; and schinifoline, *m*/*z* 258.2 → 173.1. The method validation results are summarized in Tables S5–S8.

### 3.7. Cell Culture and Culture Conditions

The HL-60 human promyelocytic leukemia cell line and the T24 human urothelial carcinoma cell line were obtained from American Type Culture Collection (Manassas, VA, USA). The LX-2 human hepatic stellate cell line was purchased from Merck (Burlington, MA, USA). The HL-60 and T24 cells were cultured in RPMI-1640 medium supplemented with 10% FBS and antibiotic-antimycotic (100 U/mL penicillin, 100 μg/mL streptomycin, and 0.25 μg/mL amphotericin B). LX-2 cells were cultured in DMEM with 2% FBS and antibiotic-antimycotic. All cells were grown in a humidified atmosphere with 5% CO_2_ at 37 °C.

### 3.8. Cell Viability Assay

Cell viability was measured by water-soluble tetrazolium salt (WST-1) assay to determine the antiproliferative activity of samples. HL-60, T24, and LX-2 cells were seeded in 24-well culture plates at a density of 4 × 10^5^, 6 × 10^3^, and 5 × 10^4^ cells/well, respectively. These cells were preincubated in medium for 24 h and then treated with different concentrations of samples (prepared in medium containing 0.1% DMSO). After incubation for 72 h, WST-1 reagent was added to each well and incubated for another 2 h. The absorbance at 450 nm was measured by a microplate reader (Thermo Scientific, Waltham, MA, USA). Cells treated with medium containing 0.1% DMSO served as a vehicle control. Doxorubicin was used as a positive control. The cell viability assay was performed with three replicates. The half-maximal inhibitory concentration (IC_50_) was calculated as the concentration of the sample that caused 50% cell growth inhibition compared with the vehicle control.

## 4. Conclusions

This study utilized an untargeted LC-MS/MS-based multi-informative molecular networking strategy to explore the antiproliferative ingredients in TR fruit. A total of 11 indole alkaloids and 47 types of quinolone alkaloids were annotated and visualized in the three targeted bioactive molecular families, and up to 25 types of quinolone alkaloids were discovered that were previously unreported in TR fruit. Four indole alkaloids and five types of quinolone alkaloids were further targeted as potentially antiproliferative compounds, and three of them can serve as the marker compounds of the most bioactive fraction F2–3. One targeted indole alkaloid (evodiamine) was confirmed to possess potent antiproliferative activity, and its structural analogues in the bioactive molecular families may serve as promising antitumor candidates for further investigation. These observations also demonstrate the advantages of the LC-MS/MS-based multi-informative molecular networking strategy in accelerating the discovery and identification of bioactive ingredients in complex natural resources such as Chinese herbal medicine.

## Figures and Tables

**Table 1:** Antiproliferative activity of different samples of *T. ruticarpum* fruit against three different human cell lines.

Sample	IC_50_ (μg/mL)		
	HL-60	T24	LX-2
50% EtOH extract	0.45 ± 0.14	3.31 ± 0.43	5.66 ± 0.24
50% MeOH extract	>20	18.68 ± 0.83	>20
MeOH extract	0.39 ± 0.05	0.54 ± 0.15	3.49 ± 1.47
EtOAc extract	0.23 ± 0.14	0.32 ± 0.27	0.47 ± 0.03
Hexane extract	0.45 ± 0.09	1.49 ± 0.15	3.56 ± 0.74
Fractions of EtOAc extract			
F1	>20	>20	>20
F2	0.80 ± 0.03	5.99 ± 0.68	8.46 ± 2.10
F2-1	5.32 ± 0.12	19.01 ± 0.26	>20
F2-2	2.77 ± 0.21	14.74 ± 0.55	14.19 ± 1.13
F2-3	0.07 ± 0.01	0.15 ± 0.05	0.39 ± 0.07
F2-4	1.98 ± 0.17	2.98 ± 0.73	2.58 ± 0.28
F2-5	1.96 ± 0.03	10.97 ± 0.78	10.43 ± 1.32
F2-6	0.51 ± 0.03	2.72 ± 0.09	2.17 ± 0.05
F3	2.70 ± 0.90	10.64 ± 1.18	>20
F4	0.73 ± 0.02	2.82 ± 0.14	2.17 ± 0.19
F5	3.25 ± 0.05	3.52 ± 0.42	7.77 ± 0.12
Doxorubicin	0.17 ± 0.06	0.13 ± 0.03	0.10 ± 0.03

Data are presented as mean ± SD (*n* = 3). IC_50_: Half maximal inhibitory concentration. Doxorubicin was used as a positive control.

**Table 2:** Characteristic compounds of the EtOAc fraction F2-3 of *T. ruticarpum* fruit in the targeted molecular families containing indole alkaloids and quinolone alkaloids.

No.	RT (Min)	Name	Molecular Formula	Calculated *m*/*z* [M+H]+	Observed *m*/*z* [M+H]+	Error (ppm)	Cosine Score ^a^	Fragment Ions (Relative Abundance in %) ^b^	Peak Area (%) ^c^	Ref. ^d^
**Indole alkaloids**										
1	6.22	Dehydroevodiamine	C_19_H_15_N_3_O	302.1288	302.1290	0.66	T: 0.996	302.1284 (4), 287.1056 (8), 286.0978 (100), 272.0810 (2), 258.1022 (3)	19.04	R; T; Yang et al. (2016) [37]
2	8.87	Evodiamine	C_19_H_17_N_3_O	304.1444	304.1440	−1.32	T: 0.988 G: 0.857	171.0920 (38), 161.0710 (22), 154.0650 (8), 144.0809 (26), 134.0603 (100), 116.0499 (55), 106.0655 (59), 91.0546 (48), 79.0546 (25), 77.0389 (21)	3.31	R; T; G; Ling et al. (2016) [40]
3	7.93	1-Hydroxyrutaecarpine	C_18_H_13_N_3_O_2_	304.1081	304.1069	−3.95	NA	304.1081 (100), 302.0919 (17), 289.0844 (31), 287.0812 (22), 261.1007 (11), 260.0814 (15), 169.0758 (20), 161.0343 (14), 136.0393 (9), 142.0650 (15)	1.29	M; S; Zhao et al. (2015) [34]; Cabral et al. (2016) [39]
4	7.69	7β-Hydroxyrutaecarpine	C_18_H_13_N_3_O_2_	304.1081	304.1076	−1.64	NA	286.0975 (100), 285.0898 (21), 258.1027 (28), 257.0949 (30), 167.0606 (43), 140.0493 (10), 130.0650 (6)	1.10	M; S; Li et al. (2016) [36]
**Quinolone alkaloids** 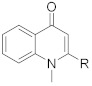 Compound name represents R group. C = Carbon; O = Oxygen attached to the carbon chain; DB = Double bond										
5	9.01	7 C (Schinifoline)	C_17_H_23_NO	258.1852	258.1848	−1.55	NA	258.1847 (7), 186.0911 (16), 173.0840 (100), 172.0752 (5), 158.0599 (14), 144.0802 (5), 132.0570 (14), 131.0492 (6)	4.21	R; Wang et al. (2013) [35]
6	8.12	11 C + 1 O +1 DB	C_21_H_29_NO_2_	328.2271	328.2262	−2.74	NA	310.2162 (20), 200.1065 (5), 187.0986 (8), 186.0915 (80), 173.0838 (100), 159.0674 (7)	3.17	M; S
7	8.84 9.01	11 C + 1 O +1 DB	C_21_H_29_NO_2_	328.2271	328.2266 328.2267	−1.52 −1.22	NA	328.2270 (69), 200.1065 (5), 186.0914 (69), 173.0838 (100), 172.0752 (5)	1.31 1.30	M; S
8	7.80 8.02	13 C + 2 O	C_23_H_35_NO_3_	374.2690	374.2678 374.2681	−3.21 −2.40	NA	356.2585 (100), 338.2481 (28), 286.1793 (6), 200.1066 (5), 186.0915 (42), 173.0838 (56)	1.16 2.32	M; S
9	7.90	15 C + 2 O + 3 DB	C_25_H_33_NO_3_	396.2533	396.2528	−1.26	NA	212.1064 (4), 200.1066 (7), 187.0985 (9), 186.0915 (100), 173.0839 (60)	1.55	M; S

^a^ The threshold of library match is cosine score ≥0.8 (a cosine score of 1 indicates perfect match; a cosine score of 0 indicates no similarity). T: TCM PCDL; G: Public spectral libraries on the GNPS website; NA: Not applicable. ^b^ The mass spectrum with the highest signal intensity was selected to represent the fragment ions if more than one molecule was annotated as the same compound (potential structural isomers). The fragment ions that are underlined are the characteristic product ions selected based on the corresponding references in the Ref. column. ^c^ Percentage of the total area of all chromatographic peaks of the EtOAc fraction F2-3. ^d^ R: Reference standard; T: TCM PCDL; G: Public spectral libraries on the GNPS website; M: Predicted by propagating the annotated structure information within the molecular family; S: Predicted by SIRIUS software. RT: Retention time.

**Table 3:** Antiproliferative activity of the targeted compounds against HL-60 and LX-2 cells.

Compound	IC_50_ (μM)	
	HL-60	LX-2
Evodiamine	0.21 ± 0.02	0.39 ± 0.02
Dehydroevodiamine	33.58 ± 1.61	80.82 ± 6.51
Schinifoline	14.83 ± 2.02	92.28 ± 7.97
Doxorubicin	0.32 ± 0.10	0.18 ± 0.05

Data are presented as mean ± SD (*n* = 3). IC_50_: Half maximal inhibitory concentration. Doxorubicin was used as a positive control.

## Data Availability

The LC-MS/MS data presented in this study are publicly available on the MassIVE website (https://massive.ucsd.edu; accessed on 16 June 2022), and the dataset accession numbers are summarized in Table S4 (Supplementary Materials).

## Supplementary Materials

The following supporting information can be downloaded at: https://www.mdpi.com/article/10.3390/molecules27144462/s1; Table S1: Summary of the annotated compounds in the targeted molecular families containing indole alkaloids and quinolone alkaloids; Table S2: Contents of evodiamine, dehydroevodiamine, and schinifoline in the F2-series fractions; Table S3: Intermediate precision of representative indole alkaloids and quinolone alkaloids in LC-Q-TOF analysis; Table S4: The accession numbers of the LC-MS/MS data of the EtOAc fractions of *T. ruticarpum* fruit on the MassIVE website (https://massive.ucsd.edu; accessed on 16 June 2022); Table S5: The precursor ion, product ion, and collision energy conditions of evodiamine, dehydroevodiamine, and schinifoline in LC-triple quadrupole analysis; Table S6: Regression data, limit of detection (LOD), and limit of quantitation (LOQ) of evodiamine, dehydroevodiamine, and schinifoline in LC-triple quadrupole analysis; Table S7: Recovery data of evodiamine, dehydroevodiamine, and schinifoline in LC-triple quadrupole analysis; Table S8: Repeatability of evodiamine, dehydroevodiamine, and schinifoline in LC-triple quadrupole analysis; Figure S1: Greenness assessment profiles of (**A**) Extraction method and (**B**) LC-MS/MS method; Figure S2: LC-MS total ion chromatogram of the EtOAc extract of *T. ruticarpum* fruit; Figure S3: LC-UV chromatogram of the EtOAc extract of *T. ruticarpum* fruit (detection wavelength at 254 nm). References [9,33,34,35,36,37,39,40,46,56] are cited in the Supplementary Materials.
