# Supplementary material for: Untargeted LC-MS/MS-Based Multi-Informative Molecular Networking for Targeting the Antiproliferative Ingredients in Tetradium ruticarpum Fruit
Source: Molecules. 2022 Jul 12;27(14):4462. doi: 10.3390/molecules27144462 (PMC9316527; doi:10.3390/molecules27144462)
Supplement: Supplementary file 1 [file molecules-27-04462-s001.zip › molecules-1803914-supplementary.pdf]

Supplementary Materials

Table S1. Summary of the annotated compounds in the targeted molecular families containing indole alkaloids and quinolone alkaloids.

No.	RT (min)	Name	Molecular formula	Calculated *m/z* [M+H]$^+$	Observed *m/z* [M+H]$^+$	Error (ppm)	Cosine score [a]	Fragment ions (relative abundance in %) [b]	Ref. [c]
**Indole alkaloids**									
1	6.22	Dehydroevodiamine	$C_{19}H_{15}N_3O$	302.1288	302.1290	0.66	T: 0.996	302.1284 (4), 287.1056 (8), 286.0978 (100), 272.0810 (2), 258.1022 (3)	R; T; Yang et al. (2016) [1]
2	8.87	Evodiamine	$C_{19}H_{17}N_3O$	304.1444	304.1440	–1.32	T: 0.988 G: 0.857	171.0920 (38), 161.0710 (22), 154.0650 (8), 144.0809 (26), 134.0603 (100), 116.0499 (55), 106.0655 (59), 91.0546 (48), 79.0546 (25), 77.0389 (21)	R; T; G; Ling et al. (2016) [2]
3	8.01	Hydroxyevodiamine	$C_{19}H_{17}N_3O_2$	320.1394	320.1381	–4.06	NA	177.0652 (7), 171.0914 (33), 150.0546 (51), 144.0801 (13), 132.0441 (32), 122.0600 (100), 95.0491 (16), 94.0646 (7), 94.0411 (26), 77.0383 (11)	M; S; Li et al. (2020) [3]
4	9.10	Rutaecarpine	$C_{18}H_{13}N_3O$	288.1131	288.1127	–1.39	T: 0.835	288.1123 (100), 286.0957 (20), 273.0888 (39), 271.0859 (26), 244.0857 (18), 169.0756 (27), 145.0390 (25), 142.0646 (17), 120.0440 (22), 115.0541 (16)	T; Ling et al. (2016) [2]
5	8.29	7,8-Dehydrorutaecarpine	$C_{18}H_{11}N_3O$	286.0975	286.0965	–3.50	NA	286.0966 (72), 285.0890 (56), 257.0939 (100), 256.0858 (26), 231.0909 (89), 167.0596 (32), 155.0599 (42), 140.0492 (26), 128.0491 (89), 77.0383 (44)	M; S; Zhao et al. (2015) [4]
6	8.09	Dihydrorutaecarpine	$C_{18}H_{15}N_3O$	290.1288	290.1276	–4.14	NA	171.0915 (100), 154.0646 (19), 144.0799 (26), 120.0438 (31), 118.0645 (6), 92.0495 (20)	M; S; Li et al. (2016) [5]
7	7.93	1-Hydroxyrutaecarpine	$C_{18}H_{13}N_3O_2$	304.1081	304.1069	–3.95	NA	304.1081 (100), 302.0919 (17), 289.0844 (31), 287.0812 (22), 261.1007 (11), 260.0814 (15), 169.0758 (20), 161.0343 (14), 136.0393 (9), 142.0650 (15)	M; S; Zhao et al. (2015) [4]; Cabral et al. (2016) [6]

8	9.01	3-Hydroxyrutaecarpine	$C_{18}H_{13}N_3O_2$	304.1081	304.1075	–1.97	NA	304.1078 (100), 302.0917 (13), 289.0842 (25), 287.0809 (17), 261.1015 (17), 259.0861 (17), 169.0765 (34), 142.0649 (16), 136.0392 (10), 115.0542 (11)	M; S; Zhao et al. (2015) [4]; Cabral et al. (2016) [6]
9	7.69	7β-Hydroxyrutaecarpine	$C_{18}H_{13}N_3O_2$	304.1081	304.1076	–1.64	NA	286.0975 (100), 285.0898 (21), 258.1027 (28), 257.0949 (30), 167.0606 (43), 140.0493 (10), 130.0650 (6)	M; S; Li et al. (2016) [5]
10	8.05	14-Formyldihydrorutaecarpine	$C_{19}H_{15}N_3O_2$	318.1237	318.1228	–2.83	NA	199.0862 (11), 171.0922 (100), 154.0652 (41), 148.0394 (37), 144.0812 (95), 120.0443 (39), 118.0651 (11), 117.0696 (9), 92.0497 (22), 77.0385 (22)	M; S; Yang et al. (2016) [1]
11	7.94	2-(1*H*-Indol-2-yl)quinazolin-4(1*H*)-one	$C_{16}H_{11}N_3O$	262.0975	262.0966	–3.43	NA	145.0393 (100), 144.0423 (16), 143.0596 (100), 120.0433 (23), 117.0440 (26), 116.0494 (41), 92.0493 (55), 90.0338 (39), 77.0380 (30), 65.0382 (22)	M; S

Quinolone alkaloids

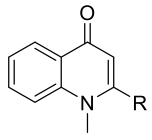

Compound name represents R group. C = Carbon; O = Oxygen attached to the carbon chain; DB = Double bond

12	5.17	6 C +1 O + 1 DB	$C_{16}H_{19}NO_2$	258.1489	258.1479	−3.87	NA	258.1474 (6), 186.0907 (27), 173.0833 (100), 172.0750 (7), 159.0673 (5), 158.0594 (11), 144.0798 (6), 132.0567 (11), 131.0486 (6)	M; S
13	9.01	7 C (Schinifoline)	$C_{17}H_{23}NO$	258.1852	258.1848	−1.55	NA	258.1847 (7), 186.0911 (16), 173.0840 (100), 172.0752 (5), 158.0599 (14), 144.0802 (5), 132.0570 (14), 131.0492 (6)	R; Wang et al. (2013) [7]
14	5.93	7 C +1 O + 1 DB	$C_{17}H_{21}NO_2$	272.1645	272.1636	−3.31	NA	272.1634 (10), 186.0909 (35), 173.0829 (100), 159.0667 (5), 158.0592 (12), 144.0797 (6), 132.0564 (8), 131.0484 (5)	M; S
15	6.71<br>11.68	8 C +1 O + 1 DB	$C_{18}H_{23}NO_2$	286.1802	286.1789<br>286.1789	−4.54<br>−4.54	NA	286.1785 (18), 186.0907 (35), 173.0833 (100), 158.0593 (6), 132.0565 (6)	M; S

16	10.74	9 C (1-Methyl-2-nonyl-4(1*H*)-quinolone)	$C_{19}H_{27}NO$	286.2165	286.2164	−0.35	G: 0.891	286.2167 (25), 186.0915 (27), 173.0845 (100), 158.0603 (9), 132.0574 (10)	G; Ling et al. (2016) [2]
17	12.39	11 C (1-Methyl-2-undecyl-4(1*H*)-quinolone)	$C_{21}H_{31}NO$	314.2478	314.2476	−0.64	G: 0.948	314.2480 (52), 186.0915 (29), 173.0840 (100), 158.0601 (5), 132.0571 (6)	G; Ling et al. (2016) [2]
18	8.44<br>11.30	11 C + 1 DB (1-Methyl-2-[(*Z*)-6-undecenyl]-4(1*H*)-quinolone and isomer)	$C_{21}H_{29}NO$	312.2322	312.2312<br>312.2326	−3.20<br>1.28	G: 0.832<br>G: 0.871	312.2322 (12), 200.1071 (6), 187.0991 (10), 186.0918 (100), 173.0837 (48), 159.0679 (10)	G; Ling et al. (2016) [2]
19	7.88<br>8.12<br>10.56	11 C + 2 DB	$C_{21}H_{27}NO$	310.2165	310.2154<br>310.2153<br>310.2158	−3.55<br>−3.87<br>−2.26	NA	187.0979 (5), 186.0913 (70), 173.0836 (100), 159.0672 (8), 158.0595 (5), 132.0566 (5)	M; S; Huang et al. (2012) [8]
20	8.28<br>8.70<br>8.94	11 C + 1 O	$C_{21}H_{31}NO_2$	330.2428	330.2419<br>330.2417<br>330.2409	−2.73<br>−3.33<br>−5.75	NA	312.2324 (100), 200.1065 (9), 187.0983 (6), 186.0916 (81), 173.0837 (81)	M; S
21	8.12	11 C + 1 O +1 DB	$C_{21}H_{29}NO_2$	328.2271	328.2262	−2.74	NA	310.2162 (20), 200.1065 (5), 187.0986 (8), 186.0915 (80), 173.0838 (100), 159.0674 (7)	M; S
22	8.84<br>9.01<br>9.31	11 C + 1 O +1 DB	$C_{21}H_{29}NO_2$	328.2271	328.2266<br>328.2267<br>328.2264	−1.52<br>−1.22<br>−2.13	NA	328.2270 (69), 200.1065 (5), 186.0914 (69), 173.0838 (100), 172.0752 (5)	M; S
23	13.18	12 C	$C_{22}H_{33}NO$	328.2635	328.2631	−1.22	NA	328.2635 (81), 186.0914 (29), 173.0837 (100), 159.0672 (5), 132.0568 (4)	M; S; Huang et al. (2012) [8]
24	14.05	13 C (1-Methyl-2-tridecyl-4(1*H*)-quinolone)	$C_{23}H_{35}NO$	342.2791	342.2794	0.88	G: 0.920	342.2795 (100), 186.0916 (28), 173.0839 (89), 158.0597 (3), 132.0569 (3)	G; Ling et al. (2016) [2]
25	12.80	13 C + 1 DB	$C_{23}H_{33}NO$	340.2635	340.2643	2.35	NA	340.2637 (39), 200.1071 (16), 187.0988 (7), 186.0917 (100), 173.0838 (62)	M; S; Ling et al. (2016) [2]
26	9.32	13 C + 2 DB	$C_{23}H_{31}NO$	338.2478	338.2474	−1.18	NA	338.2473 (13), 200.1067 (10), 187.0983 (6), 186.0914 (100), 173.0835 (49)	M; S; Ling et al. (2016) [2]
27	12.06	13 C + 2 DB	$C_{23}H_{31}NO$	338.2478	338.2476	−0.59	NA	338.2479 (8), 212.1069 (6), 200.1068 (5), 187.0992 (14), 186.0917 (100), 173.0837 (51), 159.0679 (18)	M; S; Ling et al. (2016) [2]

28	11.26	13 C + 3 DB	$C_{23}H_{29}NO$	336.2322	336.2318	−1.19	NA	212.1064 (4), 187.0984 (11), 186.0917 (100), 173.0838 (55), 159.0675 (22)	M; S
29	10.81	13 C + 4 DB	$C_{23}H_{27}NO$	334.2165	334.2157	−2.39	NA	187.0976 (11), 186.0909 (100), 184.0748 (5), 173.0830 (58), 159.0672 (22), 91.0539 (7)	M; S
30	9.66	13 C + 1 O	$C_{23}H_{35}NO_2$	358.2741	358.2737	−1.12	NA	340.2635 (100), 200.1066 (5), 187.0982 (4), 186.0916 (43), 173.0838 (45)	M; S; Ling et al. (2016) [2]
31	7.80<br>8.02	13 C + 2 O	$C_{23}H_{35}NO_3$	374.2690	374.2678<br>374.2681	−3.21<br>−2.40	NA	356.2585 (100), 338.2481 (28), 286.1793 (6), 200.1066 (5), 186.0915 (42), 173.0838 (56)	M; S
32	8.01<br>10.33	13 C + 1 O + 1 DB	$C_{23}H_{33}NO_2$	356.2584	356.2573<br>356.2581	−3.09<br>−0.84	NA	356.2586 (100), 338.2468 (7), 200.1064 (5), 187.0981 (5), 186.0912 (53), 173.0835 (79)	M; S; Huang et al. (2012) [8]
33	9.10<br>9.32	13 C + 1 O + 1 DB	$C_{23}H_{33}NO_2$	356.2584	356.2585<br>356.2579	0.28<br>−1.40	NA	338.2478 (47), 200.1070 (10), 187.0988 (9), 186.0916 (100), 173.0837 (55)	M; S; Huang et al. (2012) [8]
34	9.63	13 C + 1 O + 2 DB	$C_{23}H_{31}NO_2$	354.2428	354.2422	−1.69	NA	354.2419 (39), 284.1638 (8), 256.1685 (6), 242.1529 (6), 228.1375 (5), 200.1065 (9), 187.0981 (9), 186.0913 (100), 173.0835 (82)	M; S; Huang et al. (2012) [8]
35	14.80	14 C	$C_{24}H_{37}NO$	356.2948	356.2942	−1.68	NA	356.2951 (100), 200.1059 (2), 187.0981 (2), 186.0909 (18), 173.0837 (58)	M; S; Ling et al. (2016) [2]
36	12.16<br>12.65<br>13.52<br>16.65	14 C + 1 DB	$C_{24}H_{35}NO$	354.2791	354.2783<br>354.2783<br>354.2787<br>354.2786	−2.26<br>−2.26<br>−1.13<br>−1.41	NA	354.2780 (43), 214.1216 (20), 201.1138 (6), 200.1068 (100), 187.0982 (43), 186.0908 (6), 173.0828 (7), 172.0752 (14)	M; S; Ling et al. (2016) [2]
37	12.64	14 C + 2 DB	$C_{24}H_{33}NO$	352.2635	352.2626	−2.55	NA	352.2622 (18), 200.1062 (9), 187.0983 (7), 186.0911 (100), 173.0832 (76), 172.0748 (15), 159.0666 (9)	M; S
38	11.45	14 C + 1 O + 1 DB	$C_{24}H_{35}NO_2$	370.2740	370.2729	−3.24	NA	338.2466 (74), 200.1053 (10), 187.0986 (7), 186.0908 (100), 173.0829 (56)	M; S
39	15.35<br>17.75	15 C	$C_{25}H_{39}NO$	370.3104	370.3102<br>370.3098	−0.54<br>−1.62	NA	370.3109 (100), 186.0917 (13), 173.0838 (40)	M; S; Ling et al. (2016) [2]

40	11.03<br>14.28	15 C + 1 DB	$C_{25}H_{37}NO$	368.2948	368.2936<br>368.2942	−3.26<br>−1.63	NA	368.2953 (100), 228.1377 (5), 200.1070 (9), 187.0988 (11), <u>186.0917</u> (100), <u>173.0840</u> (66)	M; S; Ling et al. (2016) [2]
41	10.58<br>13.36	15 C + 2 DB	$C_{25}H_{35}NO$	366.2791	366.2780<br>366.2789	−3.00<br>−0.55	NA	366.2794 (28), 268.1695 (5), 200.1073 (8), 187.0994 (12), <u>186.0917</u> (100), <u>173.0838</u> (57), 159.0676 (5)	M; S; Li et al. (2020) [3]
42	10.36<br>12.53	15 C + 3 DB	$C_{25}H_{33}NO$	364.2635	364.2630<br>364.2631	−1.37<br>−1.10	NA	364.2636 (5), 200.1072 (8), 187.0992 (11), <u>186.0918</u> (100), <u>173.0838</u> (57), 159.0681 (6)	M; S; Ling et al. (2016) [2]; Ma et al. (2021) [9]
43	9.52<br>9.79<br>11.49<br>11.77<br>11.97<br>12.29	15 C + 4 DB	$C_{25}H_{31}NO$	362.2478	362.2477<br>362.2475<br>362.2471<br>362.2469<br>362.2474<br>362.2465	−0.28<br>−0.83<br>−1.93<br>−2.48<br>−1.10<br>−3.59	NA	200.1070 (7), 187.0988 (8), <u>186.0917</u> (100), <u>173.0838</u> (49), 159.0676 (5)	M; S
44	7.19<br>7.90<br>8.55<br>9.16<br>10.84	15 C + 5 DB	$C_{25}H_{29}NO$	360.2322	360.2312<br>360.2313<br>360.2310<br>360.2316<br>360.2312	−2.78<br>−2.50<br>−3.33<br>−1.67<br>−2.78	NA	200.1061 (6), 187.0977 (8), <u>186.0909</u> (100), <u>173.0829</u> (73), 159.0667 (8)	M; S
45	11.01	15 C + 1 O	$C_{25}H_{39}NO_2$	386.3054	386.3036	−4.66	NA	386.3044 (5), 368.2948 (100), 312.2311 (3), <u>186.0915</u> (18), <u>173.0837</u> (20)	M; S; Zhao et al. (2015) [4]
46	11.53	15 C + 1 O + 1 DB	$C_{25}H_{37}NO_2$	384.2897	384.2887	−2.60	NA	384.2894 (100), 366.2777 (2), <u>186.0908</u> (12), <u>173.0830</u> (20)	M; S
47	10.37	15 C + 1 O + 2 DB	$C_{25}H_{35}NO_2$	382.2741	382.2736	−1.31	NA	364.2633 (25), 212.1064 (5), 200.1064 (8), 187.0984 (11), <u>186.0915</u> (100), <u>173.0837</u> (73)	M; S
48	9.56<br>9.81	15 C + 1 O + 3 DB	$C_{25}H_{33}NO_2$	380.2584	380.2579<br>380.2578	−1.31<br>−1.58	NA	200.1065 (7), 187.0983 (9), <u>186.0915</u> (100), <u>173.0836</u> (56), 159.0672 (4)	M; S
49	7.20	15 C + 1 O + 4 DB	$C_{25}H_{31}NO_2$	378.2428	378.2416	−3.17	NA	228.1375 (5), 200.1063 (6), 187.0981 (7), <u>186.0913</u> (100), <u>173.0833</u> (44)	M; S

50	9.02	15 C + 1 O + 4 DB	$C_{25}H_{31}NO_2$	378.2428	378.2418	−2.64	NA	200.1058 (6), 187.0978 (8), 186.0909 (100), 173.0831 (61), 159.0666 (6)	M; S
51	11.18	15 C + 2 O + 1 DB	$C_{25}H_{37}NO_3$	400.2846	400.2835	−2.75	NA	356.2580 (100), 338.2465 (24), 286.1787 (5), 186.0908 (18), 173.0829 (21)	M; S
52	7.19<br>7.90<br>8.04	15 C + 2 O + 3 DB	$C_{25}H_{33}NO_3$	396.2533	396.2521<br>396.2528<br>396.2525	−3.03<br>−1.26<br>−2.02	NA	212.1064 (4), 200.1066 (7), 187.0985 (9), 186.0915 (100), 173.0839 (60)	M; S
53	9.16	15 C + 3 O + 1 DB	$C_{25}H_{37}NO_4$	416.2795	416.2784	−2.64	NA	356.2573 (100), 338.2467 (65), 286.1788 (3), 186.0904 (23), 173.0828 (27)	M; S
54	15.98	16 C	$C_{26}H_{41}NO$	384.3261	384.3256	−1.30	NA	384.3255 (100), 187.0994 (2), 186.0906 (9), 173.0833 (25), 159.0683 (2)	M; S; Ling et al. (2016) [2]
55	8.52	16 C + 2 O + 3 DB	$C_{26}H_{35}NO_3$	410.2690	410.2676	−3.41	NA	360.2306 (6), 282.1844 (8), 212.1063 (5), 200.1063 (12), 187.0981 (11), 186.0910 (100), 173.0833 (64)	M; S
56	16.57<br>18.52	17 C	$C_{27}H_{43}NO$	398.3417	398.3407<br>398.3408	−2.51<br>−2.26	NA	398.3410 (100), 186.0905 (6), 173.0829 (18)	M; S
57	15.49	17 C + 1 DB	$C_{27}H_{41}NO$	396.3261	396.3251	−2.52	NA	396.3252 (100), 200.1057 (8), 187.0977 (5), 186.0908 (44), 173.0830 (33)	M; S
58	8.75<br>9.16	17 C + 3 O + 4 DB	$C_{27}H_{35}NO_4$	438.2639	438.2626<br>438.2628	−2.97<br>−2.51	NA	378.2411 (9), 360.2302 (11), 282.1841 (8), 228.1377 (5), 212.1057 (5), 200.1066 (7), 187.0981 (9), 186.0912 (100), 173.0833 (60)	M; S

[a] The threshold of library match is cosine score ≥ 0.8 (a cosine score of 1 indicates perfect match; a cosine score of 0 indicates no similarity). T: TCM PCDL; G: Public spectral libraries on the GNPS website; NA: Not applicable.

[b] The mass spectrum with the highest library match score or signal intensity was selected to represent the fragment ions if more than one molecule was annotated as the same compound (potential structural isomers). The fragment ions that are underlined are the characteristic product ions selected based on the corresponding references in the Ref. column.

[c] R: Reference standard; T: TCM PCDL; G: Public spectral libraries on the GNPS website; M: Predicted by propagating the annotated structure information within the molecular family; S: Predicted by SIRIUS software.

RT: Retention time.

Table S2. Contents of evodiamine, dehydroevodiamine, and schinifoline in the F2-series fractions.

Fraction	Content (μg/mg, dry weight)		
	Evodiamine	Dehydroevodiamine	Schinifoline
F2	29.7 ± 2.4	19.6 ± 0.4	3.2 ± 0.1
F2-1	1.3 ± 0.1	44.7 ± 0.7	Tr
F2-2	4.5 ± 0.4	3.0 ± 0.3	Tr
F2-3	138.7 ± 3.6	92.2 ± 3.1	16.4 ± 0.4
F2-4	19.0 ± 0.4	12.1 ± 0.5	14.4 ± 0.4
F2-5	Tr	1.4 ± 0.2	Tr
F2-6	Tr	1.1 ± 0.1	Tr

Data are presented as mean ± SD (n = 3).

Tr: Trace (< 1 μg/mg).

Table S3. Intermediate precision of representative indole alkaloids and quinolone alkaloids in LC-Q-TOF analysis.

Compound	Intermediate precision [a]			
	Peak retention time		Peak area	
	Mean ± SD (min)	RSD (%)	Mean ± SD	RSD (%)
Dehydroevodiamine	6.257 ± 0.014	0.219	$1.380 \times 10^{8} \pm 0.063 \times 10^{8}$	4.598
Evodiamine	8.881 ± 0.012	0.130	$3.551 \times 10^{7} \pm 0.139 \times 10^{7}$	3.925
Schinifoline	9.017 ± 0.008	0.094	$5.134 \times 10^{6} \pm 0.168 \times 10^{6}$	3.277
Rutaecarpine	9.128 ± 0.013	0.142	$5.847 \times 10^{7} \pm 0.196 \times 10^{7}$	3.350
1-Methyl-2-nonyl-4(1*H*)-quinolone	10.748 ± 0.028	0.257	$9.382 \times 10^{7} \pm 0.325 \times 10^{7}$	3.462
1-Methyl-2-undecyl-4(1*H*)-quinolone	12.373 ± 0.027	0.215	$1.098 \times 10^{8} \pm 0.040 \times 10^{8}$	3.665
1-Methyl-2-tridecyl-4(1*H*)-quinolone	14.067 ± 0.021	0.149	$2.980 \times 10^{8} \pm 0.143 \times 10^{8}$	4.807

[a] Data obtained by using the extract of *T. ruticarpum* fruit with three replicates between three days.

Table S4. The accession numbers of the LC-MS/MS data of the EtOAc fractions of *T. ruticarpum* fruit on the MassIVE website (https://massive.ucsd.edu; accessed on 16 June 2022).

Sample	Accession number on the MassIVE website
EtOAc fraction F1	MSV000089662
EtOAc fraction F2	MSV000089663
EtOAc fraction F2-1	MSV000089664
EtOAc fraction F2-2	MSV000089665
EtOAc fraction F2-3	MSV000089666
EtOAc fraction F2-4	MSV000089667
EtOAc fraction F2-5	MSV000089668
EtOAc fraction F2-6	MSV000089669
EtOAc fraction F3	MSV000089670
EtOAc fraction F4	MSV000089671
EtOAc fraction F5	MSV000089672

Table S5. The precursor ion, product ion, and collision energy conditions of evodiamine, dehydroevodiamine, and schinifoline in LC-triple quadrupole analysis.

Compound	*m/z* value		Collision energy (V)
	Precursor ion	Product ion	
Evodiamine	304.1	134.1 [a]	30
		171.1	30
Dehydroevodiamine	302.1	286.1 [a]	40
		257.1	60
Schinifoline	258.2	173.1 [a]	30
		186.1	30

[a] Product ion with higher signal intensity used for quantification.

Table S6. Regression data, limit of detection (LOD), and limit of quantitation (LOQ) of evodiamine, dehydroevodiamine, and schinifoline in LC-triple quadrupole analysis.

Compound	Regression equation	R^2	Range (ng/mL)	LOD (ng/mL)	LOQ (ng/mL)
Evodiamine	$Y = (3.73\times10^5)X + 2.78\times10^5$	0.9992	0.5–50	0.041	0.125
Dehydroevodiamine	$Y = (2.11\times10^5)X - 8.92\times10^3$	0.9980	1–100	0.179	0.543
Schinifoline	$Y = (1.55\times10^5)X + 2.13\times10^5$	0.9997	1–100	0.240	0.727

Table S7. Recovery data of evodiamine, dehydroevodiamine, and schinifoline in LC-triple quadrupole analysis.

Compound	Original (μg/mg)	Spiked (μg/mg)	Found (μg/mg) [a]	Recovery	
				Mean ± SD (%)	RSD (%)
Evodiamine	32.18 ± 1.51	30	61.05 ± 2.61	96.23 ± 4.35	4.52
Dehydroevodiamine	20.54 ± 0.73	20	39.73 ± 1.21	95.92 ± 4.47	4.66
Schinifoline	3.23 ± 0.12	3	6.08 ± 0.25	95.10 ± 4.68	4.92

[a] Mean ± SD (n = 6); data obtained by spiking each compound into the extract of *T. ruticarpum* fruit.

Table S8. Repeatability of evodiamine, dehydroevodiamine, and schinifoline in LC-triple quadrupole analysis.

Compound	Repeatability [a]	
	Mean ± SD (μg/mg)	RSD (%)
Evodiamine	29.89 ± 1.44	4.81
Dehydroevodiamine	19.31 ± 0.59	3.07
Schinifoline	3.15 ± 0.11	3.54

[a] Data obtained by using the extract of *T. ruticarpum* fruit with six replicates on the same day.

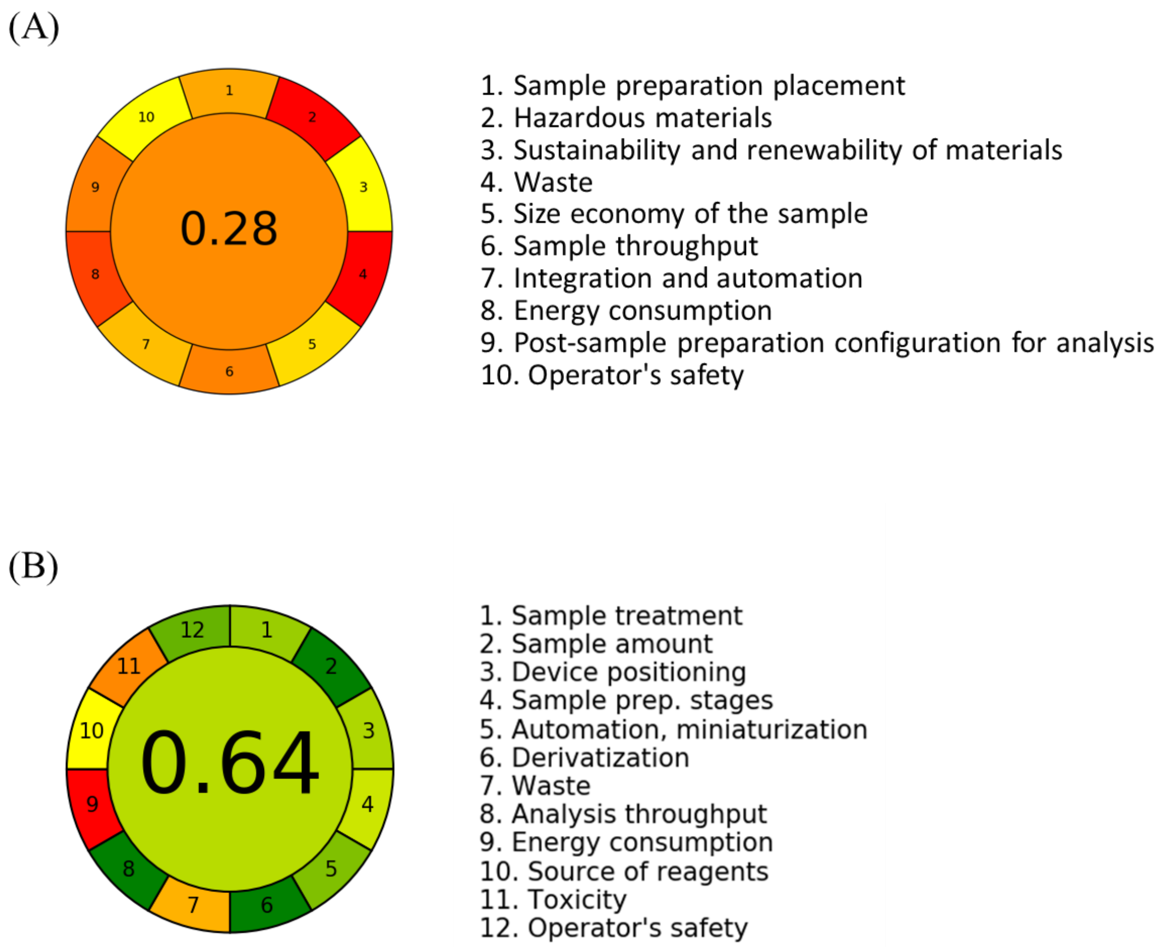

Figure S1. Greenness assessment profiles of (A) Extraction method and (B) LC-MS/MS method. The assessment was performed by using Analytical GREEnness (AGREE) software [10], and there are 10 and 12 assessment criteria for sample preparation and analytical method, respectively. The final score is ranged from zero (low greenness) to one (high greenness).

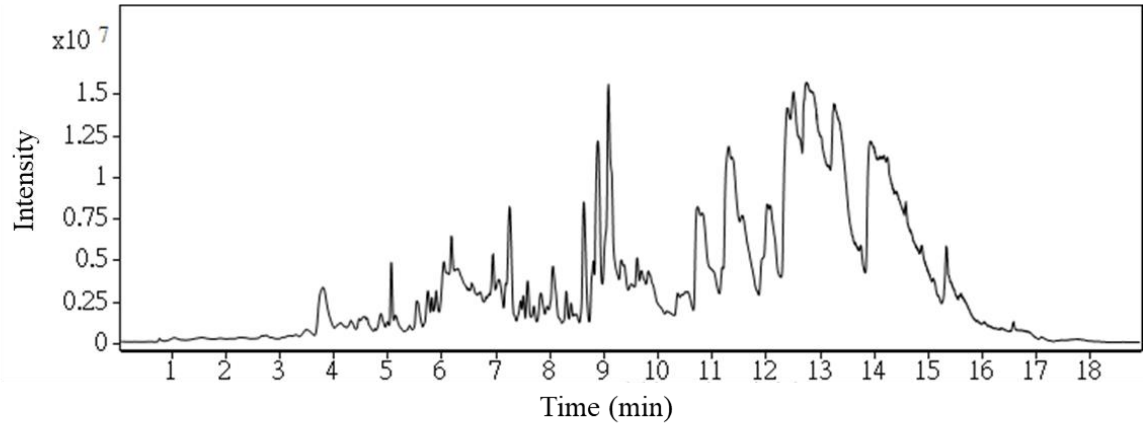

Figure S2. LC-MS total ion chromatogram of the EtOAc extract of *T. ruticarpum* fruit.

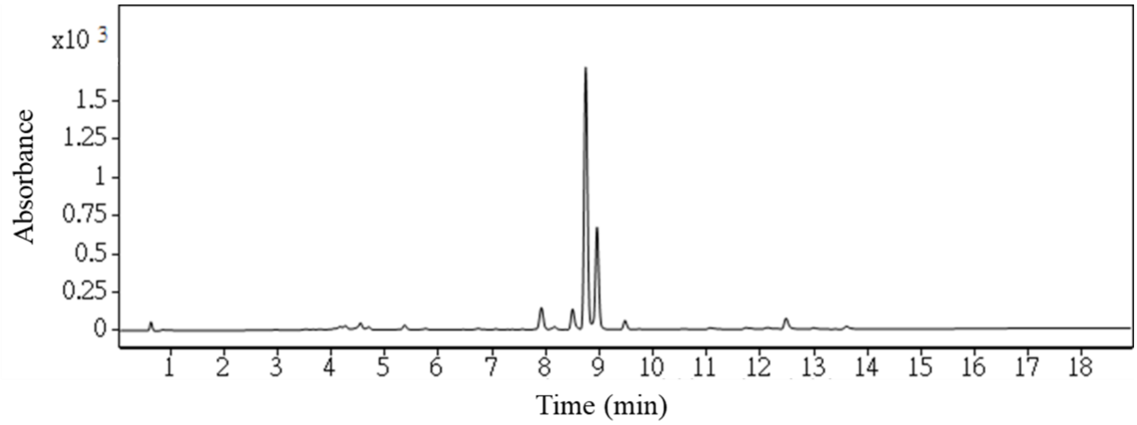

Figure S3. LC-UV chromatogram of the EtOAc extract of *T. ruticarpum* fruit (detection wavelength at 254 nm). This chromatogram was obtained by using the 1290 Infinity II LC system (Agilent Technologies) coupled to the 1260 Infinity II diode array detector (Agilent Technologies) with the same chromatographic conditions as described in the Materials and Methods (Section 3.3).

References

1. Yang, S.; Tian, M.; Yuan, L.; Deng, H.; Wang, L.; Li, A.; Hou, Z.; Li, Y.; Zhang, Y. Analysis of *E. rutaecarpa* alkaloids constituents *in vitro* and *in vivo* by UPLC-Q-TOF-MS combined with diagnostic fragment. *J. Anal. Methods Chem.* **2016**, *2016*, 4218967.
2. Ling, Y.; Hu, P.; Zhang, L.; Jin, H.; Chen, J.; Tao, Z.; Huang, L.; Ren, R. Identification and structural characterization of acylgluconic acids, flavonol glycosides, limonoids and alkaloids from the fruits of *Evodia rutaecarpa* by high performance liquid chromatography coupled to electrospray ionization and quadrupole time-of-flight mass spectrometry. *J. Chromatogr. Sci.* **2016**, *54*, 1593–1604.
3. Li, M.; Wang, C. Traditional uses, phytochemistry, pharmacology, pharmacokinetics and toxicology of the fruit of *Tetradium ruticarpum*: A review. *J. Ethnopharmacol.* **2020**, *263*, 113231.
4. Zhao, N.; Li, Z.-L.; Li, D.-H.; Sun, Y.-T.; Shan, D.-T.; Bai, J.; Pei, Y.-H.; Jing, Y.-K.; Hua, H.-M. Quinolone and indole alkaloids from the fruits of *Euodia rutaecarpa* and their cytotoxicity against two human cancer cell lines. *Phytochemistry* **2015**, *109*, 133–139.
5. Li, W.; Sun, X.; Liu, B.; Zhang, L.; Fan, Z.; Ji, Y. Screening and identification of hepatotoxic component in *Evodia rutaecarpa* based on spectrum–effect relationship and UPLC-Q-TOFMS. *Biomed. Chromatogr.* **2016**, *30*, 1975–1983.
6. Cabral, R.S.A.; Allard, P.-M.; Marcourt, L.; Young, M.C.M.; Queiroz, E.F.; Wolfender, J.-L. Targeted isolation of indolopyridoquinazoline alkaloids from *Conchocarpus fontanesianus* based on molecular networks. *J. Nat. Prod.* **2016**, *79*, 2270−2278.
7. Wang, X.-X.; Zan, K.; Shi, S.-P.; Zeng, K.-W.; Jiang, Y.; Guan, Y.; Xiao, C.-L.; Gao, H.-Y.; Wu, L.-J.; Tu, P.-F. Quinolone alkaloids with antibacterial and cytotoxic activities from the fruits of *Evodia rutaecarpa*. *Fitoterapia* **2013**, *89*, 1–7.
8. Huang, X.; Li, W.; Yang, X.-W. New cytotoxic quinolone alkaloids from fruits of *Evodia rutaecarpa*. *Fitoterapia* **2012**, *83*, 709–714.
9. Ma, C.; Liu, X.; Shan, Y.; Xu, S.; Feng, X.; Wang, Q.-Z. A new quinolone alkaloid from the fruits of *Tetradium ruticarpum*. *Nat. Prod. Res.* **2021**, *35*, 222−227.
10. Pena-Pereira, F.; Wojnowski, W.; Tobiszewski, M. AGREE—Analytical GREEnness metric approach and software. *Anal. Chem.* **2020**, *92*, 10076–10082.
